# LncRNA-NEAT1 from the competing endogenous RNA network promotes cardioprotective efficacy of mesenchymal stem cell-derived exosomes induced by macrophage migration inhibitory factor via the miR-142-3p/FOXO1 signaling pathway

**DOI:** 10.1186/s13287-020-1556-7

**Published:** 2020-01-21

**Authors:** Hanbin Chen, Wenzheng Xia, Meng Hou

**Affiliations:** 1 Department of Radiation Oncology, First Affiliated Hospital, Wenzhou Medical University, No. 2 Fuxue Lane, Wenzhou, 325000 People’s Republic of China; 2 Department of Neurosurgery, Xinhua Hospital Affiliated to Shanghai Jiaotong University School of Medicine, Shanghai, China

**Keywords:** Mesenchymal stem cells, Exosome, MIF, Cardiomyocytes apoptosis, LncRNA-NEAT1/miR-142-3p/FOXO1 signaling pathway, Oxidative stress

## Abstract

**Aims:**

Extracellular vesicles, especially exosomes, have emerged as key mediators of intercellular communication with the potential to improve cardiac function as part of cell-based therapies. We previously demonstrated that the cardioprotective factor, macrophage migration inhibitory factor (MIF), had an optimizing effect on mesenchymal stem cells (MSCs). The aim of this study was to determine the protective function of exosomes derived from MIF-pretreated MSCs in cardiomyocytes and to explore the underlying mechanisms.

**Methods and results:**

Exosomes were isolated from control MSCs (exosome) and MIF-pretreated MSCs (exosome^MIF^), and delivered to cardiomyocytes subjected to H_2_O_2_ in vitro. Regulatory long non-coding RNAs (lncRNAs) activated by MIF pretreatment were explored using genomics approaches. Exosome^MIF^ protected cardiomyocytes from H_2_O_2_-induced apoptosis. Mechanistically, we identified lncRNA-NEAT1 as a mediator of exosome^MIF^ by regulating the expression of miR-142-3p and activating Forkhead class O1 (FOXO1). The cardioprotective effects of exosome^MIF^ were consistently abrogated by depletion of lncRNA-NEAT1, by overexpression of miR-142-3p, or by FOXO1 silencing. Furthermore, exosome^MIF^ inhibited H_2_O_2_-induced apoptosis through modulating oxidative stress.

**Conclusions:**

Exosomes obtained from MIF-pretreated MSCs have a protective effect on cardiomyocytes. The lncRNA-NEAT1 functions as an anti-apoptotic molecule via competitive endogenous RNA activity towards miR-142-3p. LncRNA-NEAT1/miR-142-3p/FOXO1 at least partially mediates the cardioprotective roles of exosome^MIF^ in protecting cardiomyocytes from apoptosis.

## Introduction

According to the World Health Organization, cardiovascular diseases (CVDs) consist of a group of disorders associated with the disruption of cardiac function, and are still a major cause of morbidity and mortality in the world [1]. Since the first study reporting the ability of skeletal muscle to repair the heart in 1998, a spectrum of stem cells has been investigated for the treatment of CVDs [2, 3]. However, stem cell therapy poses several major challenges for researchers and clinicians, including poor engraftment and survival of the transplanted cells, the occurrence of ventricular arrhythmias, and the risk of tumor formation [4]. Optimizing options, including cell-free approaches, have since served as potential new therapeutic strategies that including the benefits of cell therapy, but eliminating the need for cell transplantation.

Macrophage migration inhibitory factor (MIF) is involved in multiple CVDs, including myocardial infarction [5]. MIF-knockout mice showed heart contractile dysfunction [6], and MIF deficiency overtly exacerbated abdominal aorta constriction-induced cardiac hypertrophy and contractile anomalies [7]. Furthermore, MIF pretreatment enhanced the therapeutic potential of mesenchymal stem cells (MSCs) [8], and exogenous MIF improved the paracrine effect of MSCs [9]. Given the potential therapeutic benefits of biomimetic exosomes compared with stem cells themselves, it is necessary to determine if MIF pretreatment could affect the functions of such MSC-derived exosomes.

Long non-coding RNAs (lncRNAs) are a novel class of transcripts > 200 nucleotides long with absent protein-coding potential, which regulate gene expression at the epigenetic, transcriptional, and post-transcriptional levels [10]. LncRNAs are emerging as important players in heart development, heart failure, cardiomyocyte hypertrophy, and atherosclerosis [11]. The lncRNA nuclear paraspeckle assembly transcript 1 (NEAT1) is an lncRNA localized to the nucleus, which has been demonstrated to act as an oncogene in various human cancers [12, 13]. Accumulating evidence indicates that NEAT1 serves a critical role in cell survival [14]. Furthermore, upregulation of NEAT1 contributes to treatment in doxorubicin-induced cardiac damage [15]. Exosomes can contribute to cell-to-cell communication and modulate cellular activities in recipient cells by transferring their contents, including lncRNAs, with important effects [16, 17]. However, despite the potentially important effects of lncRNAs in cells or exosomes, the impact of modulating the lncRNA component of exosomes to improve the therapeutic effect of MSC-derived exosomes remains to be explored.

Sirtuins are a family of deacetylases known to act on numerous cellular pathways [18], including the Forkhead class O (FOXO) pathway, which regulates the cellular response to oxidative stress, with cardioprotective effects [19]. The FOXO family of transcription factors serves as the integration of growth factor signaling, oxidative stress, and inflammation [20], by regulating the expression of several antioxidant enzyme genes, including SOD [21]. MiR-142-3p has also been suggested to regulate FOXO function under oxidative stress conditions [22]. FOXO transcription factors are crucial to heart homeostasis [23], and FOXO1 critically determines cardiomyocyte apoptosis, left ventricular remodeling, and ischemic oxidative stress [24, 25].

In the present study, we investigated the ability of exosomes derived from MIF-pretreated MSCs (exosome^MIF^) to counteract cardiomyocyte apoptosis induced by H_2_O_2_, and examined the mechanisms responsible for their action.

## Materials and methods

### Cell culture and treatment

#### Human-induced pluripotent stem cell (hiPSC)-derived cardiomyocytes

HiPSC-derived cardiomyocytes were obtained from Cellular Dynamics International (Madison, WI, USA) and plated and maintained according to the manufacturer’s guidelines. The cells were plated on 10 μg/mL fibronectin medium (Invitrogen 33016-015, Carlsbad, CA, USA) as a support matrix for 48 h at 37 °C and 5% CO_2_. The maintenance medium was changed every 2 days.

#### Normal human adipose-derived MSCs (ADMSCs)

ADMSCs (ATCC®) were cultured at an early passage (passage 3) in Gibco DMEM-F12 Medium, according to the supplier’s specifications, and incubated at 37 °C and 5% CO_2_. The medium was changed every 48 h. Cells were harvested using 0.05% Trypsin-EDTA (cat. number 25300-054; Gibco) at 37 °C and 5% CO_2_ for 3 min, transferred to phosphate-buffered saline (PBS), and then centrifuged at 300×*g* for 5 min. The cells were then used for the respective experiments.

#### MIF treatment

For MSC MIF treatment, cells were cultured in medium containing 100 ng/mL recombinant MIF (R&D Systems) and incubated at 37 °C for 1 h before treatment and throughout the process, as reported previously [26].

#### H_2_O_2_ treatment

H_2_O_2_ reduces cardiomyocyte viability in a concentration-dependent manner. We therefore treated cells with 100 μM H_2_O_2_ (Sigma-Aldrich, St. Louis, MO, USA) for 24 h to induce apoptosis, as reported previously [27].

### Isolation of exosomes from medium

Exosomes were isolated from the culture medium by gradient centrifugation, as reported previously [2, 28]. Following initial centrifugation for 30 min at 3000×*g*, cells and other debris were removed and the supernatant was harvested and centrifuged at 10,000×*g* for 30 min to remove microvesicles larger than exosomes. The supernatant was finally centrifuged at 110,000×*g* for 70 min. The isolation process was performed at 4 °C, and the exosomes were resuspended in PBS and stored at − 80 °C.

### Transmission electron microscopy (TEM)

TEM was performed according to a published protocol [29]. In brief, after immunoprecipitation, exosomes were stored in 1% paraformaldehyde, dehydrated via an ethanol series, and embedded in EPON. Sections (65 nm) were stained with uranyl acetate and Reynold’s lead citrate and examined with a JEM-1400plus transmission electron microscope.

### Nanoparticle tracking analysis (NTA)

The number and size of the exosomes were measured directly using a Nanosight NS 300 system (NanoSight Technology, Malvern, UK) [30]. Exosomes were resuspended in PBS at a concentration of 5 μg/mL and further diluted 100- to 500-fold to achieve 20–100 objects per frame. Samples were injected manually into the sample chamber at ambient temperature. Each sample was configured with a 488-nm laser and a high-sensitivity camera, and monitored in triplicate at a camera setting of 13 with an acquisition time of 30 s and a detection threshold setting of 7. At least 200 completed tracks were analyzed per video. The data were finally analyzed using NTA analytical software (version 2.3).

### Western blot

Exosomes and cardiomyocytes were harvested, and total protein was extracted using RIPA solution. Protein samples were denatured, separated by 10% sodium dodecyl sulfate-polyacrylamide gel electrophoresis, and transferred to polyvinylidene difluoride membranes. The membranes were blocked in 5% fat-free milk for 2 h at room temperature and then incubated with CD63 (ab59479, 1:750), CD81 (ab79559, 1:500), FOXO1 (ab39670, 1:500), and β-actin (ab179467, 1:1000) primary antibodies at 4 °C overnight. The membranes were further incubated with IgG-horseradish peroxidase goat anti-rabbit/mouse secondary antibody (ab7090/ab97040, 1:2000) for 2 h at room temperature. Signals were developed by enhanced chemiluminescence (Sigma-Aldrich). The stained protein bands were visualized using a Bio-Rad ChemiDoc XRS imaging system and analyzed using Quantity One software.

### Flow cytometric analysis of cell apoptosis

Apoptosis was determined by detecting phosphatidylserine exposure on the cell plasma membranes using an Annexin V-FITC Apoptosis Detection Kit, according to the manufacturer’s protocol. Briefly, cells were harvested, washed in ice-cold PBS, resuspended in 300 μL binding buffer, and incubated with 5 μL Annexin V-fluorescein isothiocyanate (FITC) solution for 30 min at 4 °C in dark conditions, followed by further incubation in 5 μL propidium iodide for 5 min. The cells were then analyzed immediately by bivariate flow cytometry using a BD FACSCanto II equipped with BD FACSDiva Software (Becton-Dickinson, San Jose, CA, USA).

### Calculation of caspase 3/7 and 8 activities

Caspase 3/7 and 8 activities in cardiomyocytes were determined by enzyme-linked immunosorbent assay (ELISA), as described previously [31]. Briefly, caspase 3/7 and 8 activities in cell lysates were measured using a Cell Meter Caspase 3/7, 8 Activity Apoptosis Assay Kit (AAT Bio., Sunnyvale, CA, USA) according to the user’s manual. Results were read at 520 nm in a microplate reader (Bio-Rad, Hercules, CA, USA) and expressed as fold change in activity compared with the control.

### Microarray analysis

Exosomes and cardiomyocytes were lysed immediately in 500 μL TRIzol (ThermoFisher Scientific, Waltham, MA, USA) and stored at − 80 °C before purification using a standard phenol–chloroform extraction protocol with an RNAqueous Micro Kit (ThermoFisher Scientific). The transcriptome was subjected to microarray analysis using an Affymetrix human array (ThermoFisher Scientific) and normalized based upon quantiles.

### Quantitative reverse transcription-polymerase chain reaction (qRT-PCR)

Total RNA was isolated from exosomes and cells using TRIzol (Ambion; ThermoFisher Scientific). cDNA was synthesized from 1 μg of total RNA using Superscript II reverse transcriptase, according to the manufacturer’s protocol. RT-PCR was conducted as described previously [32]. The primer sets (Invitrogen) used are listed in Table 1.

**Table 1:** Primer sequences

Genes	Sequences
LncRNA-NEAT1	F: 5′-GTACGCGGGCAGACTAACAC-3′
	R: 5′-TGCGTCTAGACACCACAACC-3′
U6	F: 5′-GCTTCGGCAGCACATATACTAAAAT-3′
	R: 5′-CGCTTCACGAATTTGCGTGTCAT-3′
miR-142-3p	F: 5′-TGTAGTGTTTCCTACTTTAT-3′
	R: 5′-GTCGTATCCAGTGCAGGG-3′
FOXO1	F: 5′-CAGCAAATCAAGTTATGGAGGA-3′
	R: 5′-TATCATTGTGGGGAGGAGAGTC-3′
GAPDH	F: 5′-TTGCCATCAATGACCCCTTCA-3′
	R: 5′-CGCCCCACTTGATTT TGGA-3′
siRNA-LncRNA-NEAT1	5′-GCCAUCAGCUUUGAAUAAAUU-3′
siRNA-LncRNA-NT	5′-UUCUCCGAA CGUGUCACGU-3′
siRNA-FOXO1	5′-CGGAGAAUGUAUACAAGCATT-3′
siRNA-NT	5′-GGAGUUAUGAGUCAGUAUATT-3′

### Small interfering (si) RNA transfection

LncRNA-NEAT1 expression in MSCs was knocked down using siRNAs, with a non-targeting siRNA as a negative control (Invitrogen). FOXO1 expression in cardiomyocytes was also knocked down by siRNAs. The procedures were conducted as described previously [9]. The target sequences are listed in Table 1. Transfection efficiency was detected by qRT-PCR and western blot.

### miR-142-3p overexpression

Cardiomyocytes were seeded into 6-well plates at a density of 1 × 10^5^ cells per well and incubated for 12 h. To induce overexpression of miR-142-3p, cells were transfected with miR-142-3p mimic or negative control (NC) mimic (Pre-miR™ miRNA Precursors, Life Technologies, Karlsruhe, Germany) using X-treme transfection reagent (Roche Applied Science, Penzberg, Germany), according to the manufacturer’s protocol. The cells were harvested for further analysis 48 h after transfection, and the transfection efficiency was analyzed by qRT-PCR.

### Luciferase reporter assay

The 3′-untranslated regions (UTR) of lncRNA-NEAT1 and FOXO1 were synthesized, annealed, and inserted into the SacI and HindIII sites of the pmiR-reporter luciferase vector (Ambion), downstream of the luciferase stop codon to induce mutagenesis of lncRNA-NEAT1 and FOXO1. The constructs were validated by sequencing. Cardiomyocytes were seeded into a 24-well plate for luciferase assay. After overnight culture, the cells were co-transfected with wild-type (WT) or mutated plasmid, and equal amounts of miR-142-3p mimic. Luciferase assays were performed using a Dual Luciferase Reporter Assay System (Promega) 24 h after transfection.

### Measurement of reactive oxygen species (ROS) production

Cells were detached from culture plates using 0.25% trypsin-EDTA, collected in 5 mL round-bottomed polystyrene tubes, and washed with 1× Wash Buffer. The cell suspension was centrifuged for 5 min at 400×*g* at room temperature, and the supernatant was discarded. The cell pellet was resuspended in 500 μL of ROS/Superoxide Detection Solution. The cells were then incubated for 30 min at 37 °C in the dark. Data were acquired using a FACScan (BD Biosciences) and analyzed using CellQuest software (BD Biosciences).

### Lipid peroxidation assay

Lipid peroxidation was measured using an assay kit (Abcam) to measure the formation of malondialdehyde (MDA), according to the manufacturer’s instructions. Briefly, fibroblasts (1 × 10^6^ cells) were homogenized on ice in 300 μL of MDA lysis buffer (containing 3 μL of 100× butylated hydroxytoluene) and then centrifuged (13,000×*g* for 10 min) to remove insoluble material. The supernatant (200 μL) was added to 600 μL of thiobarbituric acid and incubated at 95 °C for 60 min. Samples were then cooled to room temperature in an ice bath for 10 min, and the absorbance at 532 nm was measured using a spectrophotometer.

### Determination of 4-hydroxynonenal (4-HNE) levels

We evaluated 4-HNE levels using commercially available kits (Jiancheng Bioengineering Institute, Nanjing, China), according to the manufacturer’s instructions.

### Superoxide dismutase (SOD) activity

SOD activity in cells was determined using a colorimetric assay kit (Abcam), according to the manufacturer’s instructions. Briefly, protein was isolated from the cells using lysis buffer and SOD activity was measured in 10 μg of total protein extract. Absorbance was measured at 450 nm.

### Statistical analysis

Data are expressed as the mean ± standard deviation (SD). Differences between groups were tested by one-way analysis of variance, and comparisons between two groups were evaluated using Student’s *t* test. Analyses were performed using SPSS package v19.0 (SPSS Inc., Chicago, IL, USA). *P* < 0.05 was considered statistically significant.

## Results

### Exosomes derived from MSCs pretreated with MIF protected cardiomyocytes from apoptosis

**Fig. 1:**
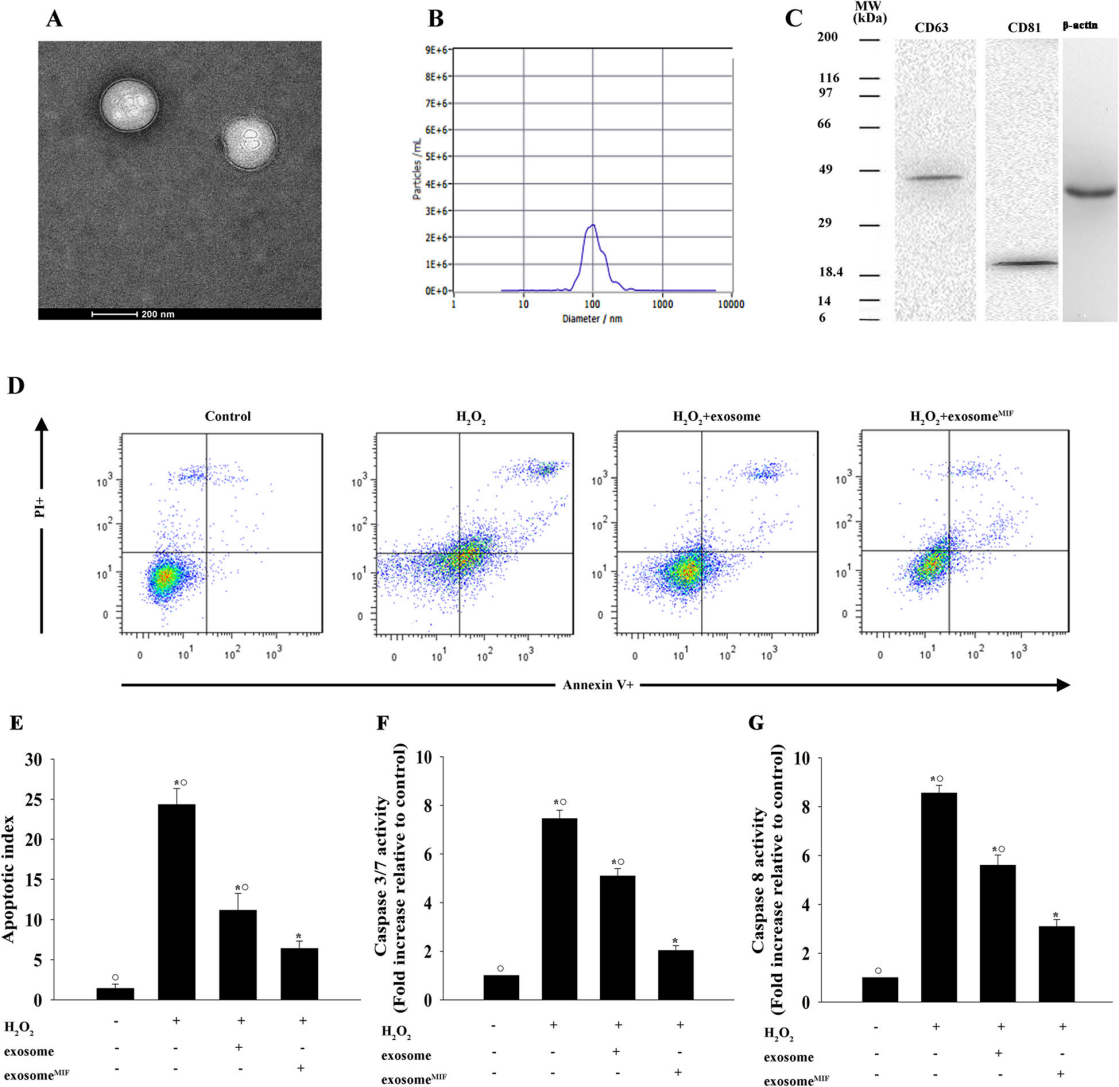
Exosomes derived from MSCs pretreated with MIF protected cardiomyocytes from apoptosis. Confirmation of exosomal collection by TEM, NTA, and western blot. **a** Representative TEM image. **b** Size range of plasma exosomes determined by NTA analysis. **c** Representative western blot images showing exosomal markers and β-actin. MSCs were treated with or without MIF. The exosomes were then collected and added to cardiomyocytes treated with H_2_O_2_. Cardiomyocytes without treatment were used as a control. **d**, **e** Representative flow cytometric dot plots of apoptotic cells after Annexin V/propidium iodide staining. Activities of **f** caspases 3/7 and **g** caspase 8 in cell lysates measured by ELISA. Each column represents mean ± SD of three independent experiments. *P < 0.05 vs. control; ^**○**^*P* < 0.05 vs. H_2_O_2_+exosome^MIF^

Given the innate cardioprotective effect of MIF, we determined if exosome^MIF^ affected human cardiomyocyte survival. Exosomes isolated from MSCs exhibited a round morphology and size of 50–100 nm, according to TEM. Moreover, expression of the exosome markers CD63 and CD81 were confirmed by western blot (Fig. 1a–c). We also determined the effects of MIF-conditioned exosomes on H_2_O_2_-induced cardiomyocyte apoptosis. Exosome^MIF^ protected cardiomyocytes from H_2_O_2_-induced apoptosis, as measured by FACS (Fig. 1d, e), and reduced caspases 3/7 and caspase 8 activities (Fig. 1f, g). However, exosomes from untreated MSCs showed less cellular protection, indicating that the observed effects were MIF-specific.

### Exosomal transfer of lncRNA-NEAT1 from MSCs to cardiomyocytes exerted cellular protection

**Fig. 2:**
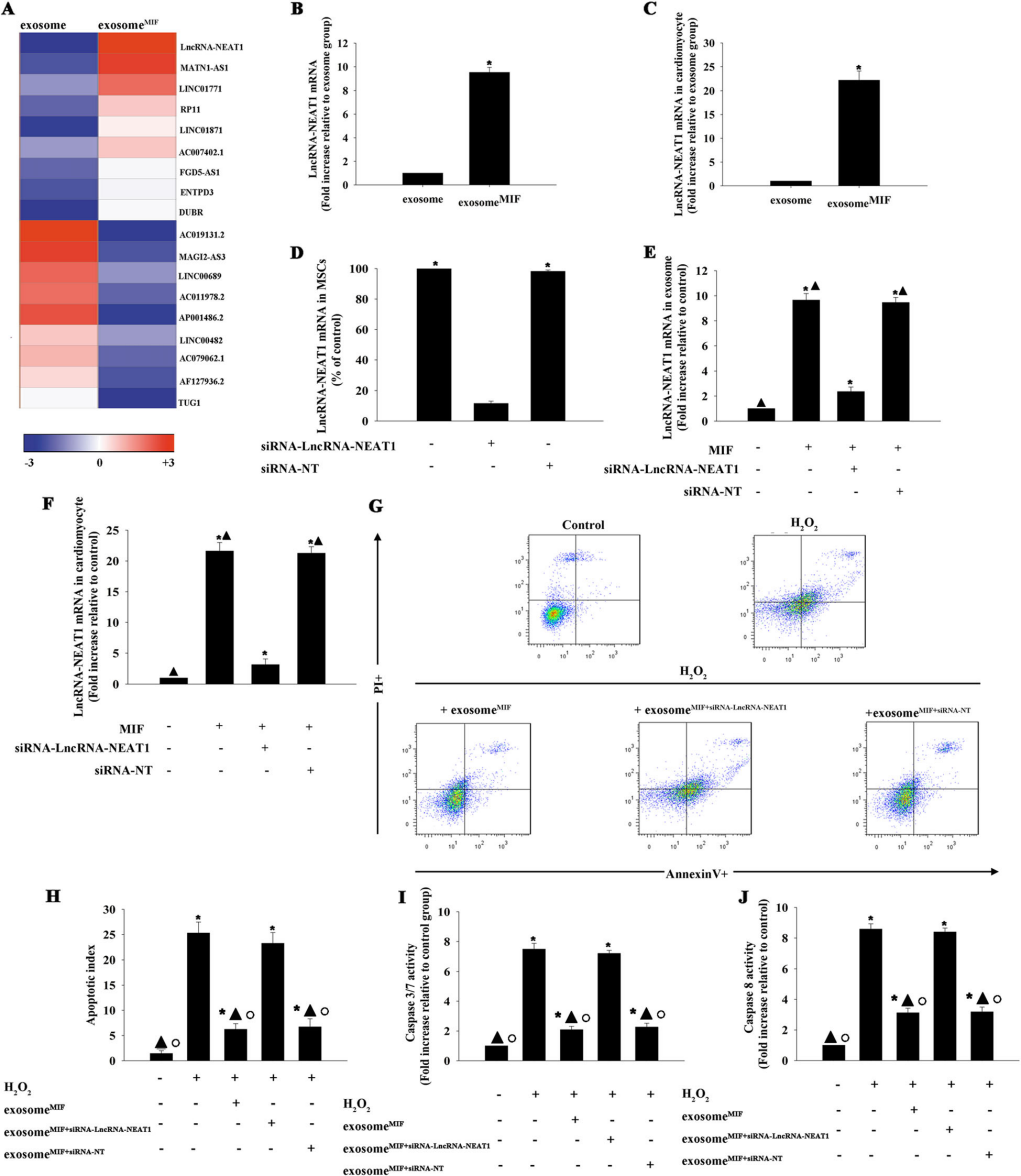
Exosomal transfer of lncRNA-NEAT1 from MSCs to cardiomyocytes and cytoprotective effect. **a** Heat map of lncRNAs differentially expressed between exosomes derived from MSCs pretreated with MIF (exosome^MIF^) and exosomes derived from untreated MSCs (exosome). **b** LncRNA-NEAT1 expression was validated by qRT-PCR in exosome^MIF^ and exosome. *P < 0.05 vs. exosome. **c** LncRNA-NEAT1 mRNA was examined by qRT-PCR in cardiomyocytes incubated with exosome^MIF^ or exosome. *P < 0.05 vs. exosome. MSCs were transfected with siRNA against lncRNA-NEAT1 or control siRNA-NT. **d** siRNA-mediated transfection efficiency was demonstrated by qRT-PCR. MSCs without treatment were used as a control. Each column represents mean ± SD of three independent experiments. *P < 0.05 vs. siRNA-lncRNA-NEAT1. **e**, **f** MSCs were transfected with siRNA against lncRNA-NEAT1 or control siRNA-NT and then treated with MIF, and the exosomes were collected and added to the cardiomyocytes. LncRNA-NEAT1 mRNA levels in exosomes and cardiomyocytes treated with the respective exosomes were examined by qRT-PCR. Exosomes derived from MSCs without treatment were used as a control. Cardiomyocytes without exosome treatment were used as a control. Each column represents mean ± SD of three independent experiments. *P < 0.05 vs. control; ^▲^*P* < 0.05 vs. MIF+siRNA-lncRNA-NEAT1. Exosomes derived from MSCs transfected with siRNA against lncRNA-NEAT1 or control siRNA-NT and treated with MIF, or with MIF alone, were added to H_2_O_2_-treated cardiomyocytes. **g**, **h** Apoptosis was analyzed by FACS. Activities of **i** caspases 3/7 and **j** caspase 8 in cell lysates were measured by ELISA. Each column represents mean ± SD of three independent experiments. *P < 0.05 vs. control; ^▲^*P* < 0.05 vs. H_2_O_2_; ^○^*P* < 0.05 vs. H_2_O_2_+exosome^MIF+siRNA-LncRNA-NEAT1^

We investigated the functional molecules responsible for the protective effect of exosome^MIF^. LncRNA expression levels in normal exosome and exosome^MIF^ were analyzed by microarray. LncRNA-NEAT1 levels were increased in exosome^MIF^, as confirmed by qRT-PCR (Fig. 2a, b), and lncRNA-NEAT1 expression levels were also significantly increased in cardiomyocytes after incubation with exosome^MIF^ (Fig. 2c). Silencing the expression of lncRNA-NEAT1 in MSCs by siRNA decreased its expression in MSCs, and in exosomes and cardiomyocytes treated with exosome^MIF^, as shown by qRT-PCR (Fig. 2d–f). Exosome^MIF^ also protected cardiomyocytes from apoptosis, as measured by FACS (Fig. 2g, h), and reduced caspases 3/7 and caspase 8 activities (Fig. 2i, j). However, this protective effect was abolished by silencing lncRNA-NEAT1 in MSCs before treatment with MIF. These results suggest that exosomes derived from MIF-treated MSCs exerted their protective effect through direct lncRNA-NEAT1 transfer.

### miR-142-3p modulated by exosomes was mediated by lncRNA-NEAT1

**Fig. 3:**
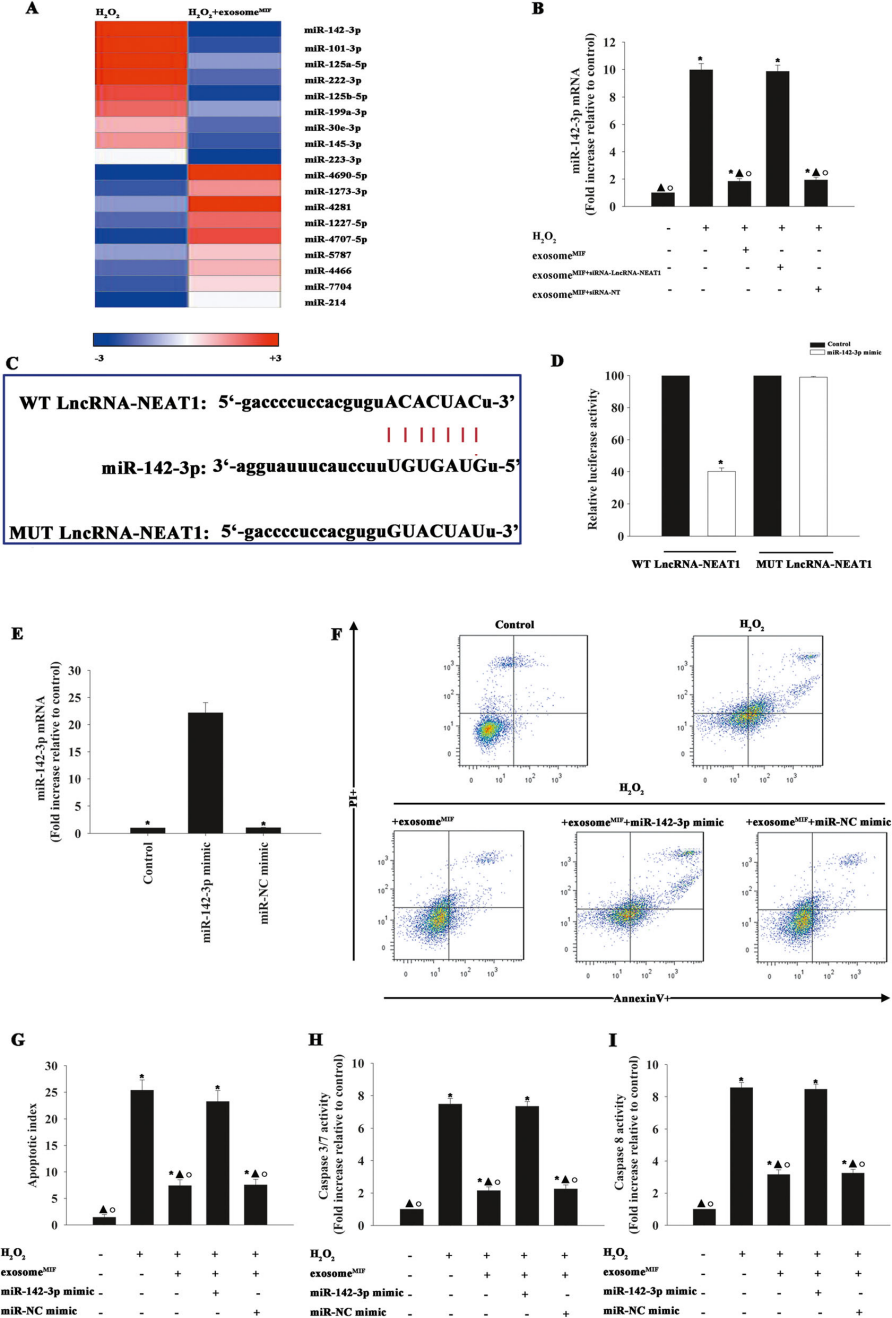
miR-142-3p modulated by exosomes was mediated by lncRNA-NEAT1. (**a**) Heat map of miRNAs differentially regulated by exosome^MIF^ in H_2_O_2_-treated cardiomyocytes. Red indicates up-regulation and blue indicates down-regulation. (**b**) qRT-PCR validation of differentially regulated miRNAs in H_2_O_2_-treated cardiomyocytes, with or without exosome^MIF^ pre-treatment. MSCs were transfected with siRNA against lncRNA-NEAT1 or control siRNA-NT, followed by MIF. The exosomes were then collected and added to cardiomyocytes treated with H_2_O_2_. Cardiomyocytes without treatment were used as a control. *P < 0.05, versus control; ^▲^*P* < 0.05 vs. H_2_O_2_; ^○^*P* < 0.05 vs. H_2_O_2_+exosomes^MIF+siRNA-LncRNA-NEAT1^. (c) Binding sites of lncRNA and miRNA. (**d**) Binding of lncRNA and miRNA was verified by dual-luciferase reporter demonstrating that miR-142-3p was a target gene of lncRNA-NEAT1. *P < 0.05, compared with control group. Cardiomyocytes were transfected with a mimic control (miR-NC mimic) or miR-142-3p mimic, followed by exosome^MIF^, and then exposed to H_2_O_2_. Cardiomyocytes with or without exosome^MIF^ were subjected to H_2_O_2_. Untreated cardiomyocytes were used as a control. (**e**) Transfection efficiency was analyzed by qRT-PCR. *P < 0.05 versus miR-142-3p mimic. (**f** and **g**) Apoptosis was analyzed by FACS. Activities of (**h**) caspases 3/7 and (**i**) caspase 8 in cell lysates were measured by ELISA. Each column represents mean ± SD of three independent experiments. *P < 0.05 vs. control; ^▲^*P* < 0.05 vs. H_2_O_2_; ^○^*P* < 0.05 vs. H_2_O_2_+exosome^MIF^+miR-142-3p mimic

To determine the potential target of lncRNA-NEAT1-induced cardiomyocyte protection, we analyzed miR expression levels in H_2_O_2_-treated cardiomyocytes and H_2_O_2_-treated cardiomyocytes exposed to exosome^MIF^ by microarray (Fig. 3a). H_2_O_2_-treated cardiomyocytes expressed higher levels of miR-142-3p, but miR-142-3p expression was substantially reduced in H_2_O_2_-treated cardiomyocytes with exosome^MIF^. However, the H_2_O_2_-induced increase in miR-142-3p was not alleviated by exosome^MIF^ derived from MSCs with silenced lncRNA-NEAT1 (Fig. 3b). A search of the bioinformatics database (LncBase) suggested the existence of a putative binding site between lncRNA-NEAT1 and miR-142-3p (Fig. 3c), which was confirmed by dual-luciferase gene reporter assay. Relative luciferase activity was significantly weakened in the lncRNA-NEAT1-WT+miR-142-3p mimic group (Fig. 3d), suggesting that miR-142-3p is a direct target of lncRNA-NEAT1. We also determined if miR-142-3p might contribute to exosome^MIF^-induced cardioprotection. MiR-142-3p overexpression (Fig. 3e) markedly increased the apoptotic percentage, caspases 3/7 and caspase 8 activities, even when exosome^MIF^ was added into H_2_O_2_-treated cardiomyocytes (Fig. 3f–i).

### Exosomal lncRNA-NEAT1/miR-142-3p protected cardiomyocytes through FOXO1 modulation

**Fig. 4:**
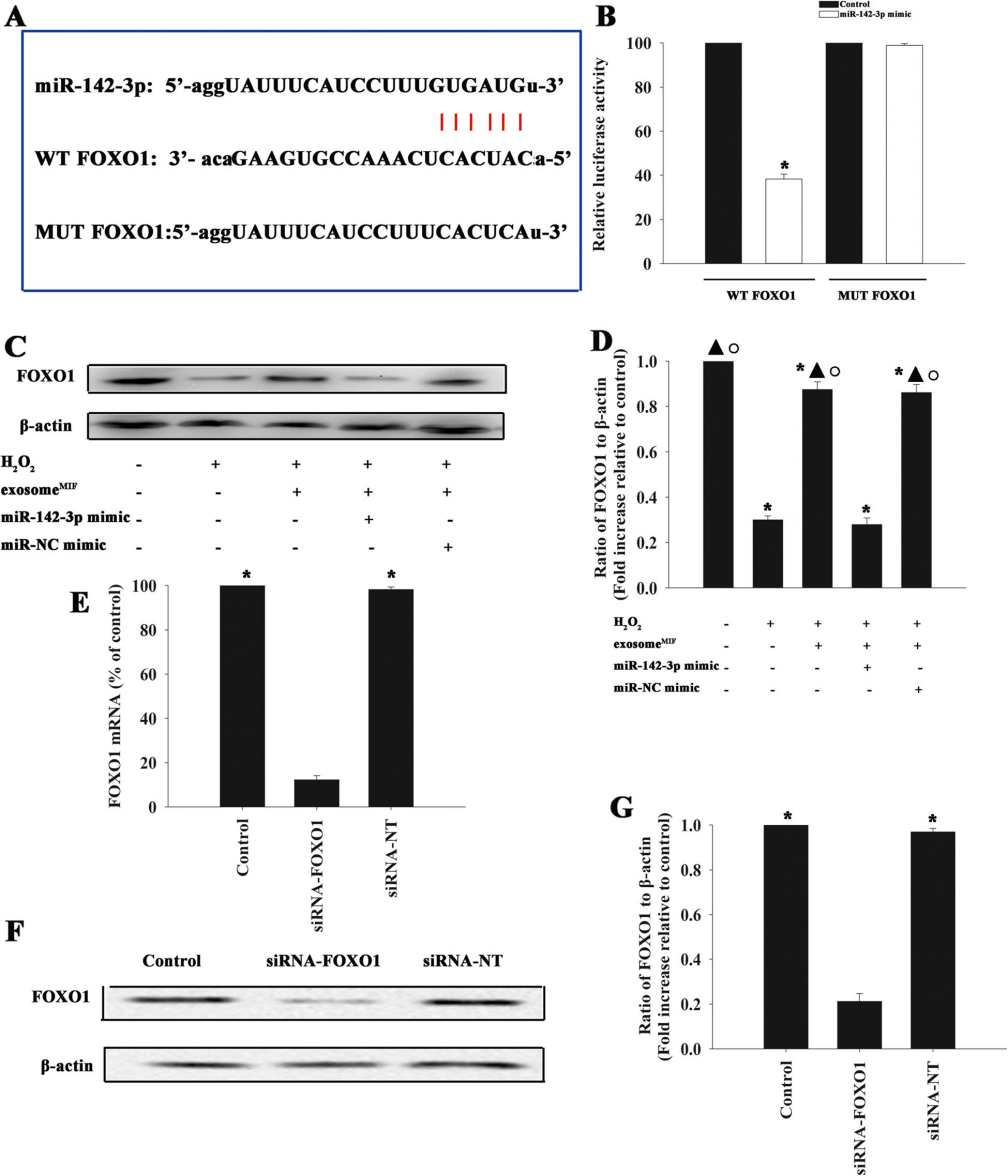
FOXO1 was a direct target of miR-142-3p. **a** Predicted binding sites between miR-142-3p and the FOXO1 3′-UTR. **b** Dual-luciferase assay was performed in cardiomyocytes after co-transfection with FOXO1 3′-UTR WT or mutant (MUT) plasmids, and miR-142-3p mimics. *P < 0.05 vs. control in the WT group. **c**, **d** Western blot analysis of FOXO1 and β-actin protein levels in cardiomyocytes transfected with mimic control or miR-142-3p mimic treated with the exosome^MIF^, and subjected to H_2_O_2_. Cardiomyocytes with or without exosome^MIF^ were subjected to H_2_O_2_. Untreated cardiomyocytes were used as a control. *P < 0.05 vs. control; ^▲^*P* < 0.05 vs. H_2_O_2_; ^○^*P* < 0.05 vs. H_2_O_2_+exosome^MIF^ miR-142-3p mimic. **e**–**g** Cardiomyocytes were transfected with siRNA-FOXO1 or siRNA-NT. Untreated cardiomyocytes were used as a control. siRNA-mediated transfection efficiency was determined by qRT-PCR (**e**) and western blotting (**f**, **g**). Each column represents mean ± SD from three independent experiments. *P < 0.05 vs. siRNA-FOXO1

**Fig. 5:**
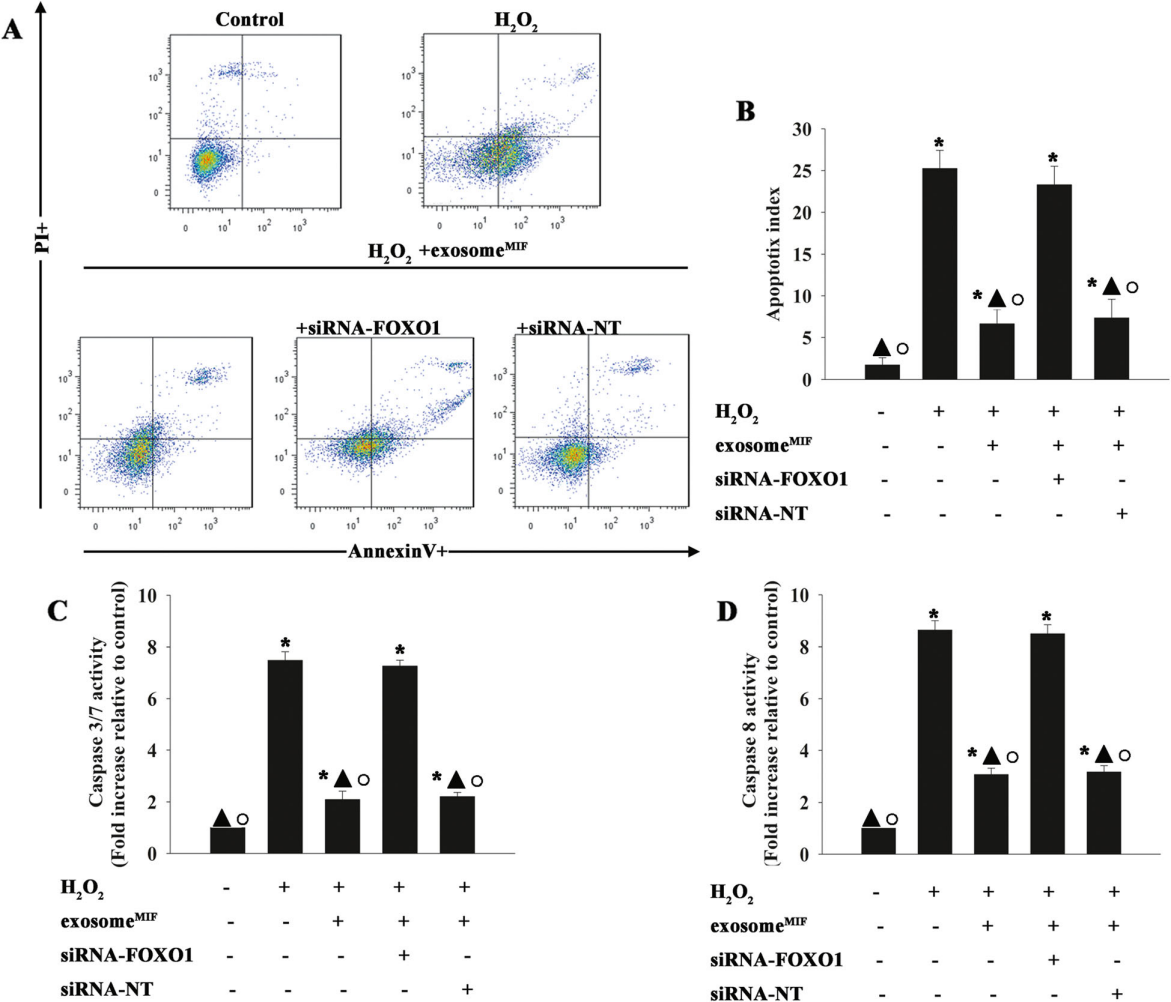
Exosome^MIF^ conferred cardiac protection by targeting FOXO1. Cardiomyocytes were transfected with siRNA-FOXO1 or siRNA-NT control, treated with exosome^MIF^, and subjected to H_2_O_2_. Cardiomyocytes with or without exosome^MIF^ were subjected to H_2_O_2_. Untreated cardiomyocytes were used as a control. (**a**) Representative distributions of propidium iodide and Annexin V staining from FACScan flow cytometric analyses of apoptotic cells. (**b**) Apoptotic cells in the above conditions. (**c**) Activities of caspases 3/7 and (**d**) caspase 8 in cell lysates were measured by ELISA. Each column represents mean ± SD of three independent experiments. *P < 0.05 vs. control; ^▲^*P* < 0.05 vs. H2O2; ^○^*P* < 0.05 vs. H_2_O_2_+exosome^MIF^+siRNA-FOXO1.

We investigated the target genes of miR-142-3p regulation using a bioinformatics database and identified a putative binding site between miR-142-3p and FOXO1 (Fig. 4a), which was confirmed by dual-luciferase gene reporter assay. Relative luciferase activity was significantly weakened in the FOXO1-WT+miR-142-3p mimic group (Fig. 4b). As expected, H_2_O_2_ treatment markedly inhibited FOXO1 expression in cardiomyocytes, and this effect was attenuated by exosome^MIF^. In addition, overexpression of miR-142-3p impaired the expression of FOXO1 (Fig. 4c, d). These results suggested that FOXO1 was a target of miR-142-3p. We further investigated the mechanism underlying the modulation of FOXO1 by exosome^MIF^ in H_2_O_2_-induced cellular apoptosis by silencing FOXO1 using siRNA. FOXO1 mRNA and protein expression levels were significantly reduced in cells transfected with siRNA-FOXO1 compared with cells transfected with non-targeting siRNA (siRNA-NT) as control (Fig. 4e–g). Exosome^MIF^ protected cardiomyocytes from apoptosis (Fig. 5a, b) and decreased the activities of caspases 3/7 and caspase 8 (Fig. 5c, d). However, these effects were abolished by silencing FOXO1, but not by transfection with the control siRNA (Fig. 5a–d). These results demonstrated that FOXO1 was a putative direct target of miR-142-3p in the cardioprotective effect of exosome^MIF^.

### Exosomes derived from MSCs pretreated with MIF modulated oxidant stress to rescue cardiomyocytes

**Fig. 6:**
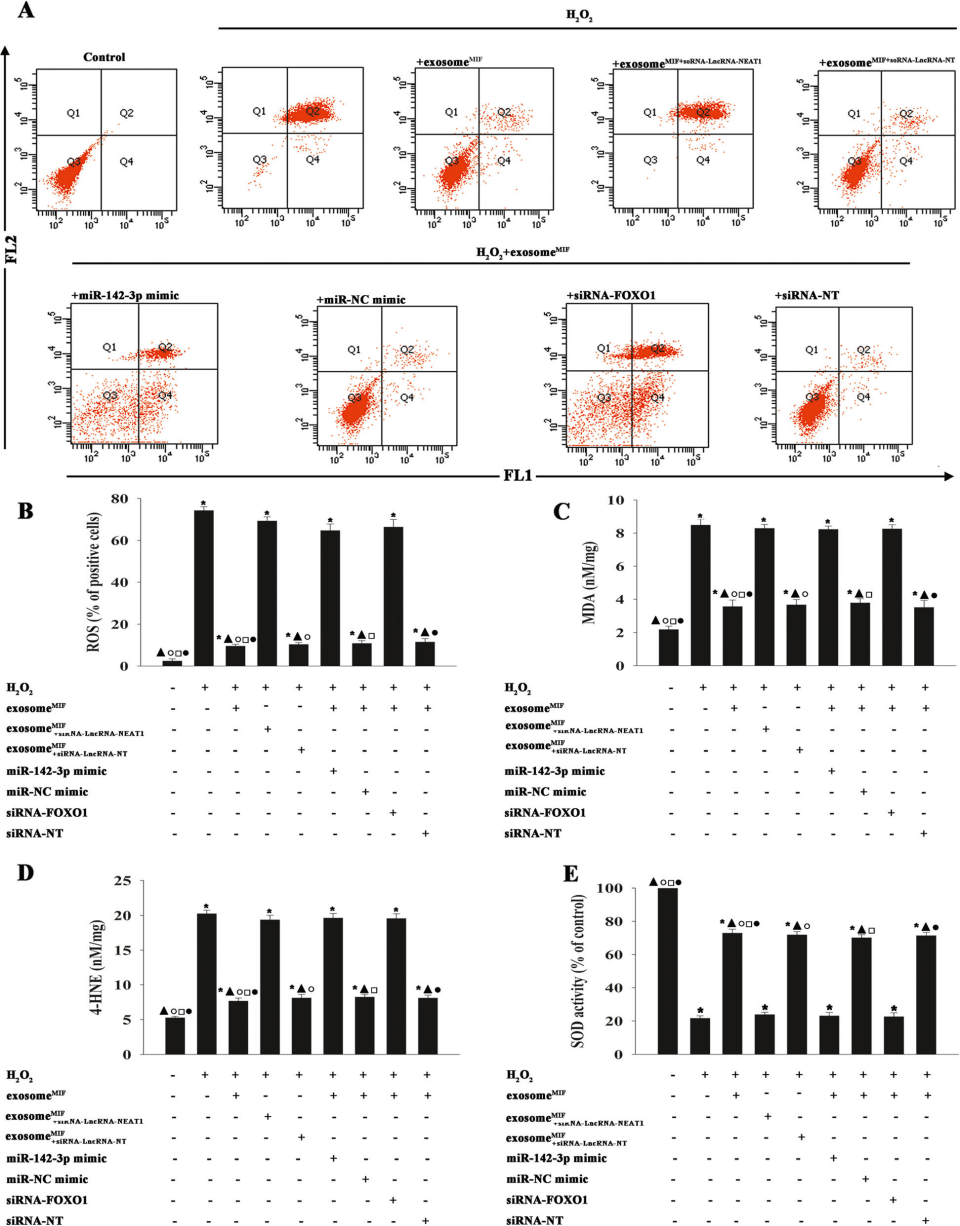
Exosomes derived from MSCs pretreated with MIF modulated oxidant stress to rescue cardiomyocytes. MSCs were transfected with siRNA against lncRNA-NEAT1 or control siRNA-NT and then treated with MIF. The exosomes were collected and added to cardiomyocytes treated with H_2_O_2_. Cardiomyocytes were transfected with miR-142-3p mimic, miR-NC mimic, siRNA-FOXO1, or siRNA-NT; treated with exosome^MIF^; and subjected to H_2_O_2_. Cardiomyocytes, with or without exosome^MIF^, were subjected to H_2_O_2_. Untreated cardiomyocytes were used as a control. **a**, **b** Intracellular ROS production was tested using a ROS detection kit and analyzed using flow cytometry. **c** Lipid peroxidation was evaluated by MDA formation. **d** Quantification of 4-HNE levels. **e** SOD activity was evaluated by colorimetric assay. *P < 0.05 vs. control; ^▲^*P* < 0.05 vs. H_2_O_2_; ^○^*P* < 0.05 vs. H_2_O_2_+exosome^MIF+siRNA-LncRNA-NEAT1^; ^□^*P* < 0.05 vs. H_2_O_2_+exosome^MIF^+ miR-142-3p mimic; ^●^*P* < 0.05 vs. H_2_O_2_+exosome^MIF^+siRNA-FOXO1

Oxidative stress is related to cellular apoptosis. We therefore investigated the feedback loop between oxidative stress and the exosomal lncRNA-NEAT1/miR-142-3p/FOXO1 signaling pathway modulated by MIF. We examined ROS generation, lipid peroxidation, and SOD activation. H_2_O_2_ significantly increased ROS generation, MDA, and 4-HNE activation (Fig. 6a–d) and decreased SOD activation (Fig. 6e), while exosome^MIF^ treatment decreased ROS generation, MDA, and 4-HNE activation (Fig. 6a–d) and increased SOD activation (Fig. 5e). These antioxidant effects of exosome^MIF^ were abolished by silencing lncRNA-NEAT1, by ectopic expression of miR-142-3p, or by silencing FOXO1 (Fig. 6a–e).

## Discussion

Myocardial disease is a leading cause of morbidity and mortality in the world [33]. Due to the limited regenerative ability of the human heart following myocardial injury, stem cell-based therapies have served as a promising approach for improvement of cardiac repair and function [34, 35]. A spectrum of stem cells has been searched for treating myocardial disease, including skeletal myoblasts, bone marrow-derived cells, induced pluripotent stem cells, endothelial progenitor cells, and cardiac progenitor cells [2]. Researchers and clinicians are also researching alternative options, including cell-free approaches, a new candidate that possesses the benefit of cell therapy without the need for cell transplantation [36, 37]. MSCs appear to be a good candidate in light of their immunosuppressive properties and paracrine ability [38, 39].

MSCs are known to elevate heart function after cardiac damages [40], and their beneficial effects are partially mediated by paracrine factors by exosome transportation [41]. Exosomes comprise functional miRNAs and lncRNAs and serve as intercellular shuttles delivering important messages to affect the gene expression and cellular functions of distant organs [42, 43]. LncRNAs in exosomes were previously shown to be tissue-specific and stage-specific, but to also be modulated by the environment [44, 45]. The current study showed that among the potential cardioprotective lncRNAs, lncRNA-NEAT1 was highly expressed in exosomes derived from MSCs treated with MIF. Moreover, exosome^MIF^ showed a better anti-apoptotic effect than exosomes from untreated MSCs in cardiomyocytes subjected to H_2_O_2_. These results support the perspective that lncRNAs in exosomes are environment-specific [46, 47]. These characteristics of lncRNAs transmitted by exosomes make them good candidate therapeutic agents.

LncRNAs are distributed in both the nucleus and cytoplasm and regulate intracellular signaling pathways via different mechanisms, including chromatin modification, gene transcription regulation, and competing endogenous RNAs (ceRNAs) [48]. Among these, ceRNAs have a potentially important role in cardiac-related diseases [49, 50]. Recent studies revealed that cardiac hypertrophy-related factor regulated cardiac hypertrophy by targeting miR-489. The lncRNA for myocardial infarction-regulatory factor inhibited autophagy by modulating miR-26a [51]. The lncRNA-NEAT1 is a 2.1-kb lncRNA transcribed from the NEAT1 gene, constituting a nuclear body with multiple roles in gene expression [52]. NEAT1 is important for RNA stability [53]. MiR-142-3p was found to be inhibited by lncRNA-NEAT1 in the current study, and previous studies have reported multiple functions of miR-142-3p in CVD [54, 55]. Forced expression of miR-142 induced extensive apoptosis and cardiac dysfunction, while loss of miR-142 fully rescued cardiac function in a murine heart failure model [56]. In the present research, the lncRNA-NEAT1/miR-142-3p axis mediated the effect of exosome^MIF^ in protecting cardiomyocytes from apoptosis. Furthermore, the protection of cardiomyocytes in vitro by exosome^MIF^ was markedly abolished by silencing lncRNA-NEAT1 expression in MSCs or by miR-142-3p overexpression in cardiomyocytes. These support the important role of exosomal lncRNA-NEAT1/miR-142-3p targeting in exosome^MIF^-mediated cardiovascular protection.

The FOXO1 transcription factor plays a role in cardiac metabolic flexibility and cell survival [57]. FOXO1 was previously shown to be a pivotal factor in cardiac autophagy, and restored FOXO1-related autophagy protected against cardiac aging and recovered mitochondrial integrity [58]. Modest FOXO1 overexpression was recently shown to be cardioprotective, by maintaining cardiac proteostasis and ameliorating age-associated functional decline [59]. Consistent with these findings, the present study showed that exosome^MIF^ enhanced FOXO1 activity in cardiomyocytes via lncRNA-NEAT1/miR-142-3p to exert its cytoprotective effect.

Previous studies have suggested many complex molecular events resulting in the progression of cardiac damage [60]. Among these causes, oxidative stress and the associated inflammatory responses are likely to be the major signaling cascades causing cardiac damage [61]. Cardiac infarction leads to a significant decrease in the levels of antioxidant enzymes such as SOD, and an increase in 4-HNE adducts, resulting in cardiomyocyte apoptosis [62, 63]. LncRNA-NEAT1 was recently shown to be involved in a protective mechanism against neuronal injury through modulating oxidative stress [64]. The activation of FOXO transcription factors in response to oxidative stress can induce a wide range of genes that regulate cellular responses such as resistance to cellular apoptosis [21]. Our current results showed that exosome^MIF^ treatment attenuated oxidative stress through regulating the expression of several antioxidant enzyme genes, including SOD, to induce the ROS scavenging process, while silencing lncRNA-NEAT1, miR-142-3p overexpression, or siRNA-FOXO1 abolished the antioxidant effect of exosome^MIF^ treatment.

## Conclusion

The present study demonstrated that exosomes derived from MIF-treated MSCs prevented H_2_O_2_-induced cardiomyocyte apoptosis, and that these beneficial effects were mediated by the novel exosome/lncRNA-NEAT1/miR-142-3p/FOXO1 pathway. These results therefore identified a novel ceRNA signaling pathway to optimize stem cell-based cardioprotective functions. Given that exosomes are easy to obtain, exosome-mediated therapy represents a potentially useful approach for clinical applications.

## Data Availability

All data and materials are available in the manuscript.

## Abbreviations

MIF: Macrophage migration inhibitory factor
MSCs: Mesenchymal stem cells
lncRNAs: Long non-coding RNAs
miR: MicroRNA
CVDs: Cardiovascular diseases
FOXO1: Forkhead class O 1
hiPSC: Human-induced pluripotent stem cell
hADMSCs: Human adipose-derived MSCs
TEM: Transmission electron microscopy
NTA: Nanoparticle tracking analysis
qRT-PCR: Quantitative reverse transcription-polymerase chain reaction
siRNA: Small interfering RNA
ROS: Reactive oxygen species
MDA: Malondialdehyde
4-HNE: 4-Hydroxynonenal
SOD: Superoxide dismutase
