# Correction to: LncRNA-NEAT1 from the competing endogenous RNA network promotes cardioprotective efficacy of mesenchymal stem cell-derived exosomes induced by macrophage migration inhibitory factor via the miR-142-3p/FOXO1 signaling pathway

**DOI:** 10.1186/s13287-020-01898-y

**Published:** 2020-09-03

**Authors:** Hanbin Chen, Wenzheng Xia, Meng Hou

**Affiliations:** 1 Department of Radiation Oncology, First Affiliated Hospital, Wenzhou Medical University, No. 2 Fuxue Lane, Wenzhou, 325000 People’s Republic of China; 2 Department of Neurosurgery, Xinhua Hospital Affiliated to Shanghai Jiaotong University School of Medicine, Shanghai, China

**Correction to: Stem Cell Res Ther 11, 31 (2020)**

**https://doi.org/10.1186/s13287-020-1556-7**

Following publication of the original article [1], the authors identified an error in Table 1. The primer of human LncRNA-NEAT1 should be revised to F: 5′ - GTGGCTGTTGGAGTCGGTAT − 3′ and R: 5′ - ACCACGGTCCATGAAGCATT − 3′.

**Table 1:** Primer sequences

Genes	Sequences
LncRNA-NEAT1	F: 5′ - GTGGCTGTTGGAGTCGGTAT − 3′
	R: 5′ - ACCACGGTCCATGAAGCATT − 3’
U6	F: 5′- GCTTCGGCAGCACATATACTAAAAT − 3’
	R: 5′- CGCTTCACGAATTTGCGTGTCAT − 3’
miR-142-3p	F: 5′ - TGTAGTGTTTCCTACTTTAT − 3′
	R: 5′ - GTCGTATCCAGTGCAGGG − 3’
FOXO1	F: 5′ - CAGCAAATCAAGTTATGGAGGA − 3′
	R: 5′ - TATCATTGTGGGGAGGAGAGTC − 3’
GAPDH	F: 5′- TTGCCATCAATGACCCCTTCA − 3’
	R: 5′- CGCCCCACTTGATTT TGGA − 3’
siRNA-LncRNA-NEAT1	5′- GCCAUCAGCUUUGAAUAAAUU − 3’
siRNA-LncRNA-NT	5′- UUCUCCGAA CGUGUCACGU − 3’
siRNA-FOXO1	5′- CGGAGAAUGUAUACAAGCATT − 3’
siRNA-NT	5′- GGAGUUAUGAGUCAGUAUATT − 3’

The correct table has been included in this correction.
